# Copy number variation of *FCGR* genes in etiopathogenesis of sarcoidosis

**DOI:** 10.1371/journal.pone.0177194

**Published:** 2017-05-04

**Authors:** Marlena Typiak, Krzysztof Rębała, Agnieszka Haraś, Monika Skotarczak, Jan Marek Słomiński, Anna Dubaniewicz

**Affiliations:** 1 Department of Pulmonology, Medical University of Gdansk, Gdansk, Poland; 2 Department of Forensic Medicine, Medical University of Gdansk, Gdansk, Poland; 3 2nd Department of Radiology, Medical University of Gdansk, Gdansk, Poland; Peking University First Hospital, CHINA

## Abstract

We have previously revealed that, in contrast to polymorphism of *FCGR2B* and *FCGR3B*, polymorphism of *FCGR2A*, *FCGR2C* and *FCGR3A* genes, encoding receptors for Fc fragment of immunoglobulin G (Fcγ receptors), play a role in increased level of circulating immune complexes with occurrence of *Mycobacterium tuberculosis* heat shock proteins in patients with sarcoidosis. However, this immunocomplexemia might also be caused by decreased clearance by immune cells due to a changed copy number of *FCGR* genes. Thus, the next step of our study was to evaluate copy number variation of *FCGR2A*, *FCGR2B*, *FCGR2C*, *FCGR3A* and *FCGR3B* in this disease. The analysis was carried out by real-time quantitative PCR on 104 patients and 110 healthy volunteers. Despite previously detected variation in allele/genotype frequencies of *FCGR* in sarcoidosis and its particular stages, there was no copy number variation of the tested genes between sarcoidosis or its stages and healthy control, as well as between stages themselves. A relevant increase in copy number of *FCGR2C* and *FCGR3B* in Stage IV of sarcoidosis vs. other stages and controls was detected, but this observation was based on a limited number of Stage IV patients. Hence, polymorphism of *FCGR* genes seems to be more important than their copy number variation in etiopathogenesis of sarcoidosis in patients from the Polish population.

## Introduction

Sarcoidosis (SA) is a multisystem, granulomatous disorder with unknown etiology. In genetically predisposed hosts, infectious, non-infectious factors, and autoimmunity are considered in etiopathogenesis of SA. Due to clinical, radiological and histopathological characteristics similar to tuberculosis, *Mycobacterium tuberculosis* (Mtb) and its antigens, e.g. heat shock proteins (Mtb-hsps), have been often suggested as potential cause of SA [1–5]. Mtb-hsps are involved in formation of immune complexes (ICs) and may be crucial in connecting infection and autoimmunity, which are both considered in sarcoidosis [3,4,6,7]. In our SA patients, we have found an increased concentration of circulating immune complexes with high levels of Mtb-hsps, especially Mtb-hsp16, a marker of a dormant stage of *M*. *tuberculosis* [6,7]. The high level of ICs in blood of our SA patients suggests presence of antigenemia, which may be a result of persistent occurrence of phagocyted mycobacteria with increased release of Mtb-hsps, which are presented to T and B lymphocytes activating cellular and humoral immune responses [3–7]. However, the increased level of ICs may be as well a result of altered elimination of antigens and ICs due to dysfunction of receptors for Fc fragment of immunoglobulin G (FcγR) from class II and III, important in phagocytosis and clearance of immune complexes [8,9]. Hence, we have analyzed expression of FcγRs on monocytes and phagocytic activity of these cells in our SA patients, revealing an increased number of FcγRII+ and FcγRIII+ monocytes with their higher phagocytic activity, which however did not eliminate circulating ICs [10]. It could point to lowered affinity of FcγRs to ICs due to polymorphism of *FCGR* genes, encoding Fcγ receptors from classes II and III (*FCGR2A*, *FCGR2B*, *FCGR2C*, *FCGR3A*, *FCGR3B*), and/or aberration in their copy number (CN), which has been revealed in many other autoimmune disorders, but not yet in SA. Therefore, we have analyzed polymorphism of these genes in our SA patients and revealed that, in contrast to polymorphism of *FCGR2B* and *FCGR3B*, polymorphism of *FCGR2A*, *FCGR2C* and *FCGR3A* may contribute to immunocomplexemia present in sarcoidosis [11,12]. Moreover, since an aberrated number of copies (CN ≠ 2) of *FCGR* genes would lead to disruption in the presence of FcγRs on immune cells, causing up- or down-regulation of an (auto)immune response, in the current study we performed the first in the world analysis of copy number variation (CNV) of *FCGR2A*, *FCGR2B*, *FCGR2C*, *FCGR3A* and *FCGR3B* genes in an ethnically homogenous, Caucasian group of patients with sarcoidosis and healthy controls.

## Materials and methods

### Study groups

The study was performed in accordance with the Declaration of Helsinki. Ethical approval for the study was granted by the Independent Bioethics Committee for Scientific Researches, Medical University of Gdansk, Poland (NKEBN/337/2009). Written informed consents were obtained from every participant of the study.

### Patients with sarcoidosis

Caucasian patients with sarcoidosis were recruited and observed from 2007 to 2014, every patient for at least three years to ensure acquisition of data about possible recurrences of the disease or its chronic character (follow-up duration from three to eight years, average four years). A number of 104 untreated patients (56 smokers, 48 non-smokers) with newly diagnosed pulmonary sarcoidosis at pulmonology hospitals in Gdansk, Poland, were included in the study (Table 1). Diagnosis of SA was based on histological (scalenobiopsy of the lymph nodes), clinical and radiological evidences. Stages of the disease were identified on the basis of radiological evidence (high resolution computed tomography) according to widely approved classification, proposed by Scadding [13]. Thirty one patients were classified to Stage I of sarcoidosis (bilateral hilar lymphadenopathy), forty nine patients to Stage II (bilateral hilar lymphadenopathy and diffuse pulmonary infiltrations), eighteen to Stage III (diffuse pulmonary infiltrations), and six to Stage IV (fibrosis and cavities) of the disease. Twenty patients had Löfgren’s syndrome. There was no statistically significant difference in age (U Mann-Whitney test: p≥0.060 for all comparisons), gender (Fisher’s exact test: p≥0.232 for all comparisons) or presence of Löfgren’s syndrome (Fisher’s exact test: p≥0.162 for all comparisons) between the stages of sarcoidosis in the tested group of patients.

**Table 1:** Comparative characteristics of patients with pulmonary sarcoidosis (SA) and healthy individuals (Contr.). The numbers in parentheses indicate percentage of individuals with a certain parameter.

Parameter	SA n = 104 (100%)	Contr. n = 110 (100%)
**Age: mean [years]**	41	42
**Age: range [years]**	21–68	18–79
**Female**	41 (39%)	50 (45%)
**Male**	63 (61%)	60 (55%)
**BCG vaccination**	104 (100%)	110 (100%)
**Positive PPD skin test**	0	0
**Relapses**	0	0
**Cough**	49 (47%)	0
**Dyspnoea**	10 (10%)	0
**Fever**	18 (17%)	0
**Night sweats**	2 (2%)	0
**Weight loss**	5 (5%)	0
**Erythema nodosum**	20 (19%)	0
**Arthritis**	20 (19%)	0

Microbiological and cytological examination of the lymph nodes and sputum samples revealed no acid-fast bacilli (PCR, culture of the *M*. *tuberculosis* strain), fungi or atypical cells.

### Controls

Caucasian healthy individuals were recruited from 2007 to 2014. The control group consisted of 110 unrelated healthy volunteers originating from the same geographic area as the group of patients. No statistically significant differences in the gender distribution (Fisher’s exact test: p = 0.270) and in the age of the enrolled individuals (U Mann-Whitney test: p = 0.060) were revealed between the two groups (Table 1). The control group also did not differ from the SA patients in percentage of smokers (59 smokers, 51 non-smokers; Fisher’s exact test: p = 1.000).

All the individuals showed normal results of chest radiographs, blood and serum analysis, as well as no acid-fast bacilli in sputum smears and in the sputum culture of the *M*. *tuberculosis* strain.

None of the controls or SA patients had a familial history of tuberculosis, sarcoidosis or autoimmune diseases. All participants of the study were not infected with HIV.

### Methods

DNA was extracted from peripheral blood samples with the use of a non-enzymatic method and quantified spectrophotometrically [14]. Copy number variation of *FCGR* genes was analysed in a 7900HT Fast Real-Time PCR System (Applied Biosystems) with the use of pre-designed TaqMan Copy Number Assays for *FCGR2A* (Hs00103511_cn), *FCGR2B* (Hs00134082_cn), *FCGR3A* (Hs00139300_cn) and *FCGR3B* (Hs04211858_cn), and a Custom TaqMan Copy Number Assay for *FCGR2C* (FCGR2C_CC5IPK0) from Life Technologies. Detailed information on the used assays is enclosed in Supplementary Information (S1 File). The reaction was carried out in the presence of 10 ng of DNA and a TaqMan Copy Number Reference Assay for RNase P. All the samples were analysed in quadruplicate. The copy number of the tested genes was determined with the use of CopyCaller Software v2.0 (Applied Biosystems). Fisher’s exact test was applied for comparison of frequencies of detected copy number variants between patients with sarcoidosis (including different stages of the disease) and control subjects. Fisher’s exact test was also used to compare a cumulative copy number of Fcγ receptor genes activating immune response (*FCGR2A*, *FCGR2C*, *FCGR3A* and *FCGR3B*) as well as a cumulative copy number of the activating Fcγ receptor genes reduced by a copy number of *FCGR2B* gene inhibiting immune response. As far as several DNA samples showed failed amplification at one or more *FCGR* genes, they were not considered in statistical evaluation of association of respective genes with the disease. Accordingly, analysis of co-occurrence of different CNs of *FCGR* genes was performed on 103 patients with sarcoidosis and 103 healthy controls with complete genotypes for five CNVs.

## Results

Frequencies of copy numbers of particular *FCGR* genes for controls and patients as well as for all stages of the disease are presented in Table 2. In contrast to *FCGR2A* and *FCGR2B* genes, copy number variation of *FCGR2C*, *FCGR3A* and *FCGR3B* genes was found in both analyzed groups. Copy number variation in *FCGR2A* and *FCGR2B* genes was noted only in one (0.93%) and three (2.78%) healthy individuals, respectively. On the other hand, CNV of *FCGR2C* gene was present in 45.19% of SA patients and 41.67% of healthy individuals. CNV of *FCGR3A* gene occurred in 13.46% of SA patients and 18.18% of healthy controls, whereas CNV of *FCGR3B* gene was present in 29.13% of patients with sarcoidosis and in 22.43% of the controls.

**Table 2:** Frequencies of CNs of the studied Fcγ receptor genes, observed in healthy controls and in patients with sarcoidosis, including different stages of the disease.

Gene	CN	Controls	Sarcoidosis	Stage I	Stage II	Stage III	Stage IV
*FCGR2A*	2	0.991	1.000	1.000	1.000	1.000	1.000
	< 2	0.000	0.000	0.000	0.000	0.000	0.000
	> 2	0.009	0.000	0.000	0.000	0.000	0.000
	N	107	104	30	47	16	6
*FCGR2B*	2	0.981	1.000	1.000	1.000	1.000	1.000
	< 2	0.000	0.000	0.000	0.000	0.000	0.000
	> 2	0.028	0.000	0.000	0.000	0.000	0.000
	N	108	104	30	47	16	6
*FCGR2C*	2	0.589	0.548	0.667	0.489	0.563	0.333
	< 2	0.336	0.365	0.300	0.426	0.375	0.167
	> 2	0.084	0.087	0.033	0.085	0.063	0.500*
	N	108	104	30	47	16	6
*FCGR3A*	2	0.841	0.865	0.900	0.830	0.938	1.000
	< 2	0.178	0.125	0.100	0.149	0.063	0.000
	> 2	0.009	0.010	0.000	0.021	0.000	0.000
	N	110	104	30	47	16	6
*FCGR3B*	2	0.776	0.702	0.700	0.660	0.875	0.500
	< 2	0.065	0.096	0.100	0.106	0.063	0.000
	> 2	0.159	0.192	0.200	0.213	0.063	0.500**
	N	107	103	30	46	16	6

* P < 0.05 for comparison with healthy controls and with stages I, II, III of sarcoidosis; P < 0.01 for comparison with combined stages I+II+III of sarcoidosis

** P < 0.05 for comparison with stage III of sarcoidosis

N: total number of tested individuals

There were no statistically significant differences in copy number variation of *FCGR2A*, *FCGR2B*, *FCGR2C*, *FCGR3A* and *FCGR3B* genes between SA or its stages and healthy controls, as well as between the stages themselves. We have detected only a relevant increase in CN of *FCGR2C* and *FCGR3B* in the last Stage IV of sarcoidosis vs. other stages and controls, but this observation was based on a limited number of six patients classified to Stage IV of SA. There was a significant increase of CN>2 of *FCGR2C* gene in Stage IV of SA versus controls (50.0% vs. 8.3%, p = 0.015), versus all the other patients with SA (50.0% vs. 6.5%, p = 0.009) and versus Stages I, II and III analysed separately (50.0% vs. 3.3%, 8.5% and 6.3%, respectively; p = 0.010, p = 0.025 and p = 0.046, respectively). We have also found an increase of CN>2 of *FCGR3B* gene in Stage IV vs. Stage III of SA (50.0% vs. 6.3%, p = 0.046.

Additionally, a significant increase in a total count of copies of *FCGR2A*, *FCGR2C*, *FCGR3A* and *FCGR3B* genes was revealed in Stage IV of SA versus healthy controls (on the average 8.8 vs. 7.6 copies, respectively; p = 0.048). Stage IV of SA also showed a significant increase in the total count of copies of *FCGR2A*, *FCGR2C*, *FCGR3A* and *FCGR3B* genes reduced by a copy number of *FCGR2B* gene in comparison to healthy subjects (on the average 6.8 vs. 5.6 copies, respectively; p = 0.043).

Frequencies of CN genotypes of the studied Fcγ receptor genes, observed in healthy controls and in patients with sarcoidosis, are presented in Table 3. In both analysed groups a typical genotype with two copies of every *FCGR* gene was the most frequent (48.5% in SA patients, 49.5% in controls). The second most frequent genotype was the one with one copy of *FCGR2C* gene (13.6% in SA patients, 16.5% in controls). Other frequently observed genotypes were the ones with simultaneous deletion of neighbouring *FCGR3A* and *FCGR2C* genes, and simultaneous deletion or duplication of neighbouring *FCGR2C* and *FCGR3B* genes.

**Table 3:** Frequencies of CN genotypes of the studied Fcγ receptor genes, observed in healthy controls and in patients with sarcoidosis.

CN genotype*	Controls	Sarcoidosis
2,0,0,2,2	0.010	–
2,1,0,2,2	–	0.010
2,1,0,3,2	0.010	0.010
2,1,1,2,2	0.010	0.019
2,1,1,3,2	0.078	0.049
2,1,2,2,2	0.058	0.039
2,2,0,1,2	–	0.010
2,2,0,2,2	0.010	0.019
2,2,1,1,2	0.058	0.078
2,2,1,2,2	0.165	0.136
2,2,1,3,2	0.010	0.039
2,2,2,1,2	0.010	–
2,2,2,2,2	0.495	0.485
2,2,2,3,2	0.010	0.019
2,2,3,3,2	0.058	0.068
2,2,4,4,2	–	0.010
2,3,2,1,2	–	0.010
2,3,3,2,2	0.010	–
3,2,3,2,4	0.010	–
N	103	103

* CN values corresponding to *FCGR2A*, *FCGR3A*, *FCGR2C*, *FCGR3B* and *FCGR2B* genes, respectively, following their order in the *FCGR* locus on chromosome 1q23.3

Raw data on CN counts for the tested *FCGR* genes for healthy controls and patients with sarcoidosis are presented in supplementary information (S2 File).

## Discussion

In the current study copy number variation was shown for *FCGR2C*, *FCGR3A* and *FCGR3B* genes in both tested and control group, which is in agreement with analysis of CNV at the *FCGR* locus [15]. Variation in copy number of *FCGR2A* and *FCGR2B* genes was noted only in several individuals.

We have found a lack of association between copy number of *FCGR2A*, *FCGR2B*, *FCGR2C*, *FCGR3A*, *FCGR3B* genes and risk of development of sarcoidosis in our patients. An association that was significant in the present study concerns increased copy number of *FCGR2C* and *FCGR3B* genes in Stage IV of SA. An increased copy number (CN>2) of *FCGR2C* gene was found in Stage IV of SA in comparison to healthy controls, to all the other patients with SA and to Stage I, II and III of the disease analyzed separately. Additionally, an increase in the copy number (CN>2) of *FCGR3B* gene has been shown in Stage IV versus Stage III of SA. Furthermore, in Stage IV of SA vs. healthy controls, a higher total count of copies of *FCGR2A*, *FCGR2C*, *FCGR3A* and *FCGR3B* genes activating immune response was revealed, as well as a higher total count of copies of these genes reduced by a copy number of *FCGR2B*.

To the best of our knowledge, this is the first analysis of copy number variation (CNV) of *FCGR2A*, *FCGR2B*, *FCGR2C*, *FCGR3A* and *FCGR3B* genes in sarcoidosis and its particular stages, which are considered as separate disease entities by some authors. Studies of this genetic variation have been performed in many other autoimmune disorders, e.g. systemic lupus erythematosus (SLE), rheumatoid arthritis (RA), granulomatosis with polyangiitis (GPA; formerly Wegener’s granulomatosis) and idiopathic thrombocytopenic purpura (ITP), but not yet in sarcoidosis [16–19].

In the studies of genetic predisposition to develop autoimmune disorders, CNV was proven only for *FCGR2C*, *FCGR3A* and *FCGR3B*, but not for *FCGR2A* and *FCGR2B* genes [8]. Additionally, it has been reported that *FCGR2C* and *FCGR3B* genes are incorporated into the most frequently occurring copy number variation region (CNR) in the *FCGR* locus—CNR1 [16]. It results in common presence of copy number variation in one of these genes, if a CNV is detected in the other [15,16]. This finding is in agreement with our present study, presenting relevant changes in CN of *FCGR2C* and *FCGR3B* genes in Stage IV of SA. It is also supported by conjoined presence of aberrated copy numbers (CN<2 or CN>2) of *FCGR2C* and *FCGR3B* genes, presented in Table 3. Results of our present study are also in agreement with the lack of association between CNV of *FCGR3A* gene and etiology of ulcerative colitis, Kawasaki disease, RA in Caucasian population of the United Kingdom, as well as Caucasian and Chinese patients with SLE [16,20–22]. In parallel to the results of our analysis in sarcoidosis and its Stages I, II, and III, no copy number variation of *FCGR3B* gene has been observed by some researchers in SLE, RA, anti-neutrophil cytoplasmic antibody-associated vasculitis (AASV), primary Sjögren’s syndrome (pSS), Addison’s disease, Grave’s disease and anti-glomerular basement membrane antibody disease (anti-GBM disease) [16,18,23–26].

Furthermore, in agreement with our analysis of CNV of *FCGR2C* gene in patients with the most severe Stage IV of sarcoidosis, an increased copy number of this gene, corresponding to an elevated number of functional, open reading frame 57Q variant of *FCGR2C*, has been revealed in idiopathic thrombocytopenic purpura [19]. Both the 57Q allele of *FCGR2C* gene, resulting in a formation of a functional FcγRIIc receptor on immune cells and initiation of an (auto)immune response, as well as an increased copy number of *FCGR* genes are so-called high-responder genetic variants of FcγRs. The occurrence of high-responder variants is linked to chronic inflammatory disorders with inappropriate leukocyte activation, expanding tissue damage in the affected organs [8]. An increased CN of *FCGR3B* gene, observed in our patients with chronic Stage IV of SA, is also included to high-responder genetic variants. Its occurrence in our patients with the last Stage IV of SA is in agreement with observations of increased CN of *FCGR3B* gene in patients with IPF, patients of Spanish ancestry with SLE and primary Sjögren’s syndrome, British patients with AASV and Han Chinese patients with psoriasis vulgaris [8,27–29]. Moreover, the presence of an elevated total count of copies of *FCGR* genes encoding receptors that activate immune response (*FCGR2A*, *FCGR2C*, *FCGR3A*, *FCGR3B*) in Stage IV of SA vs. healthy individuals is consistent with elevated immune response occurring in this most advanced stage of the disease. This observation is also significant after decreasing this count by a number of *FCGR2B* gene copies, that encodes the only Fcγ receptor which inhibits initiation of immune response after its induction by an (auto)antigen.

However, in autoimmune disorders, which are connected with high levels of circulating immune complexes, like in our patients in Stages I/II of SA, the so-called low-responder genetic variants of FcγRs have been reported to be overrepresented [8,30]. The group of low-responder genetic variants includes i.a. a low copy number of *FCGR* genes, resulting in a diminished immune response and disrupted clearance of ICs [8]. In contrast to the lack of association between CNV of the tested *FCGR* genes and development of Stages I-III of SA in our patients, a decreased CN of *FCGR3A* gene has been revealed in Taiwanese patients with RA and SLE, although also an increased CN was shown to be a risk factor for the disease development in the same patients with SLE [31]. Other authors have found a decreased CN of *FCGR3B* gene in SLE, RA, GPA, microscopic polyangiitis, AASV, pSS and systemic sclerosis [16–18,24,32,33]. However, in Kawasaki disease (KD) a low copy number of *FCGR3B* gene was considered as protective factor against the disease development, pointing to an increased CN of this gene as a risk factor for KD [21].

In the above mentioned autoimmune disorders, as well as in Stage IV of sarcoidosis, an increased copy number of *FCGR2C* and *FCGR3B* genes, leads to a higher expression of activating FcγRIIc and FcγRIIIb receptors on immune cells [16]. This is in agreement with previously shown elevated presence of both FcγR class II and III receptors on monocytes in peripheral blood of our patients with sarcoidosis [10]. Moreover, in our previous study we have reported an increase in frequency of functional 57Q allele and 57XQ genotype of *FCGR2C* gene in Stage III and IV of SA [12]. In contrast to a 57X allele, creating a stop codon, presence of 57Q allele of *FCGR2C* results in production of a full-length, functional FcγRIIc receptor on a surface of immune cells. Therefore, the increased copy number of *FCGR2C* gene in Stage IV of sarcoidosis might correspond in a great proportion to the functional 57Q variant of the gene, leading to increased FcγRIIc expression and accelerated activation of monocytes, macrophages, neutrophils, NK cells and B lymphocytes [19,34]. Activation of these cells results in phagocytosis of ICs and/or bacteria, (auto)antigen presentation, oxidative burst, antibody-dependent cell-mediated cytotoxicity and/or antibody production [19,34]. Subsequently it results in migration of monocytes, macrophages, neutrophils and lymphocytes to the site of inflammation with their proliferation, and may result in formation of a sarcoid granuloma [10].

Elevated copy number of *FCGR3B* gene, encoding FcγRIIIb, in Stage IV of SA adds to this sequence of events. FcγRIIIb is expressed mostly on neutrophils, on which it is the most common Fcγ receptor. In concordance with high expression of FcγRIIIb and higher clearance of ICs in our patients with chronic Stage IV than in earlier stages of sarcoidosis, the increased copy number of *FCGR3B* gene was found to correspond with elevated FcγRIIIb presence on neutrophils, increased uptake of immune complexes in serum and higher extravasation of neutrophils into tissues to clear deposited ICs [10,16,30]. Additionally, since FcγRIIIb is bound to a cell membrane only with a glycosylphosphatidylinositol anchor, it needs to colocalise with and signal through other receptors, such as FcγRIIa, in order to trigger neutrophils’ phagocytosis, oxidative burst and formation of neutrophil extracellular traps in tissues, a proinflammatory process that is linked to autoimmunity [35]. In our previous study, we have shown increased frequency of 131HH genotype of *FCGR2A* gene, encoding FcγRIIa, in more advanced stages of SA versus initial Stages I/II [12]. The possession of 131HH genotype results in higher affinity of FcγRIIa receptor to immune complexes and initiation of augmented (auto)immune response.

Taken together, the increased copy number of *FCGR2C* and *FCGR3B* genes, and higher frequency of functional 57Q and 57XQ variants of *FCGR2C*, as well as 131HH genotype of *FCGR2A* in our SA patients with Stage IV can trigger elevated phagocytosis of ICs by neutrophils, monocytes and macrophages with prolonged intracellular persistence of bacteria or antigens, e.g. Mtb-hsp, in phagocytes, following excessive antigen presentation to T and B lymphocytes, their activation and increased proliferation with formation of a sarcoid granuloma. Additionally, an increased CN of *FCGR2C* in a chronic Stage IV of SA in comparison to Stages I-III, as well as an increased CN of *FCGR3B* in comparison to Stage III, may be a cause of irreversible fibrosis of lung parenchyma and serve as a prognostic marker in sarcoidosis. However, due to a limited number of patients in the most severe Stage IV of SA, available for the study, further analyses are needed. Furthermore, in the light of the previously reported polymorphism of *FCGR2A*, *FCGR2C* and *FCGR3A* genes in our patients with SA, as well as the lack of association between CNV of *FCGR2A*, *FCGR2B*, *FCGR2C*, *FCGR3A* and *FCGR3B* genes in Stages I, II and III of SA, and in all patients with SA analyzed together, polymorphism of *FCGR* genes seems to have a greater impact on genetic predisposition to develop sarcoidosis than copy number variation of these genes.

## Supporting information

S1 File Detailed information on the used TaqMan Copy Number Assays (Life Technologies). (DOC)

S2 File Data set of copy number counts of *FCGR2A*, *FCGR2B*, *FCGR2C*, *FCGR3A* and *FCGR3B* genes, detected in healthy volunteers (Controls) and patients with sarcoidosis (Sarcoidosis). (XLSX)
