# Supplementary material for: Copy number variation of FCGR genes in etiopathogenesis of sarcoidosis
Source: PLoS One. 2017 May 4;12(5):e0177194. doi: 10.1371/journal.pone.0177194 (PMC5417662; doi:10.1371/journal.pone.0177194)
Supplement: S1 File — (DOC) [file pone.0177194.s001.doc]

**Detailed information on the used TaqMan Copy Number Assays (Life Technologies)**

*Copy number variation of FCGR genes was analysed with the use of pre-designed TaqMan Copy Number Assays for FCGR2A, FCGR2B, FCGR3A and FCGR3B genes, and a Custom TaqMan Copy Number Assay for FCGR2C gene.*

| Gene | Assay ID | Location of the detected gene fragment | Context sequence* | Genome location** | Amplicon length [nt] |
| --- | --- | --- | --- | --- | --- |
| *FCGR2A* | Hs00103511_cn | exon 1 - intron 1 | TCCCAGAAACCTGTGGCTGCTTCAA | Chr.1:161505516 | 90 |
| *FCGR2B* | Hs00134082_cn | intron 9 | CCCTTCTCCCCTGTTGCCTTTTCTG | Chr.1:161677405 | 78 |
| *FCGR2C* | FCGR2C_CC5IPK0 | intron 2 - exon 3 | ACCTCCTCTCTCTGCCCCTCAGCAG | Chr.1:161559342 | 105 |
| *FCGR3A* | Hs00139300_cn | intron 3 | GTCTGAAGTCTGGCAAGGGAGCCCC | Chr.1:161549907 | 106 |
| *FCGR3B* | Hs04211858_cn | intron 5 | AGGAGAACTAACTCAATGTAAACAT | Chr.1:161626930 | 101 |

*Context sequence is a nucleotide sequence for which a TaqMan probe is designed

**Genome location is a location of a middle nucleotide of the context sequence
